# Interaction between gut microbiota and sex hormones and their relation to sexual dimorphism in metabolic diseases

**DOI:** 10.1186/s13293-023-00490-2

**Published:** 2023-02-07

**Authors:** Jose Antonio Santos-Marcos, Marina Mora-Ortiz, Manuel Tena-Sempere, Jose Lopez-Miranda, Antonio Camargo

**Affiliations:** 1 Lipids and Atherosclerosis Unit, Department of Internal Medicine, Reina Sofia University Hospital, 14004 Córdoba, Spain; 2 Department of Medical and Surgical Sciences, University of Córdoba, 14004 Córdoba, Spain; 3 Maimónides Biomedical Research Institute of Córdoba (IMIBIC), Av. Menendez Pidal, s/n., 14004 Córdoba, Spain; 4 Department of Cell Biology, Physiology, and Immunology, University of Córdoba, 14004 Córdoba, Spain; 5 CIBER Fisiopatologia de la Obesidad y la Nutricion (CIBEROBN), Instituto de Salud Carlos III, 28029 Madrid, Spain

**Keywords:** Gut microbiota, Sex steroids, Sex differences, Obesity, Metabolic syndrome, Type 2 diabetes

## Abstract

Metabolic diseases, such as obesity, metabolic syndrome (MetS) and type 2 diabetes (T2D), are now a widespread pandemic in the developed world. These pathologies show sex differences in their development and prevalence, and sex steroids, mainly estrogen and testosterone, are thought to play a prominent role in this sexual dimorphism. The influence of sex hormones on these pathologies is not only reflected in differences between men and women, but also between women themselves, depending on the hormonal changes associated with the menopause. The observed sex differences in gut microbiota composition have led to multiple studies highlighting the interaction between steroid hormones and the gut microbiota and its influence on metabolic diseases, ultimately pointing to a new therapy for these diseases based on the manipulation of the gut microbiota. This review aims to shed light on the role of sexual hormones in sex differences in the development and prevalence of metabolic diseases, focusing on obesity, MetS and T2D. We focus also the interaction between sex hormones and the gut microbiota, and in particular the role of microbiota in aspects such as gut barrier integrity, inflammatory status, and the gut–brain axis, given the relevance of these factors in the development of metabolic diseases.

## Highlights

- Accumulating evidences show that the alterations in the gut microbiota associated to metabolic diseases are different in men and women, and these differences may influence sex differences in the development and prevalence of metabolic diseases.
- The key aspects involved in these pathologies include lipopolysaccharide-inflammation, gut barrier integrity, gut microbiota-derived metabolites and gut–brain axis.
- Sex steroids, mainly estrogen and testosterone, are thought to play a prominent role in the sexual dimorphism of gut microbiota.
- The influence of sex hormones is reflected both in men and women, and among women themselves due to hormonal changes associated with the menopause.
- The interaction between sex steroids and the gut microbiota plays a prominent role in the development of metabolic diseases.

## Introduction

The increasing incidence of metabolic diseases, and in particular obesity, MetS, and T2D, in the world population, has made these pathologies a serious health, social, and economic problem [1–3]. Interestingly, these pathologies show a marked sexual dimorphism in their development and prevalence, with a clear influence of sex hormones [4]. The overall prevalence of obesity is higher in women than in men, as women are more likely to gain abdominal fat with age. In fact, the prevalence of visceral obesity associated with MetS is currently much higher in women in many regions of the world. Moreover, the prevalence of T2D is reversed by life stage, with more men having diabetes before puberty and more women having diabetes after menopause. It is interesting, in this regard, to observe the pattern of body fat distribution, given its key role in metabolic diseases. Two patterns of fat distribution have been described, an abdominal (visceral) pattern, typical of men and postmenopausal women, and a peripheral (subcutaneous) pattern, typical of premenopausal women [5, 6]. Both patterns, which have a genetic basis and are regulated by sex steroid hormones [7], are related to the development of metabolic diseases, with central fat distribution showing a pathological profile [8] versus a protective profile of peripheral fat [9].

The influence of sex hormones on metabolic diseases is supported by conditions in which their normal level is altered. Both transgender men and women show fat redistribution after sex steroid treatment [10]. The hormonal changes of menopause also lead to fat redistribution [11], as well as an increased risk of T2D [12], while hormone therapy with estrogens and progestogens in postmenopausal women reduces its incidence [13]. Androgen deprivation therapy in men with prostate cancer results in increased fat mass [14], higher prevalence of MetS [15] and elevated risk of T2D [16], while testosterone treatment decreases visceral fat in nonobese aging men with symptoms of androgen deficiency and low-normal serum testosterone levels [17]. In addition, testosterone replacement improves insulin sensitivity and glycemic control, patients with hypogonadism suffering T2D and MetS, partially through reducing central obesity [18]. Polycystic ovary syndrome (PCOS) is a multifactorial disorder with various genetic, endocrine and environmental abnormalities [19]. Considerable genetic heterogeneity underlies PCOS, as several genes’ variants have been linked to this disorder. Moreover, women with PCOS present hyperandrogenism which has been associated to increased central adiposity [20] and increased risk of MetS [21]. Oophorectomy-induced estrogen depletion in postmenopausal women increases the risk of T2D [22].

In recent years, a sexual dimorphism in the composition of the gut microbiota has also been highlighted [23] in which sex hormones seem to play a prominent role [24]. In fact, a growing body of scientific evidence indicates that the interaction between the gut microbiota and its host is key to the development of metabolic diseases [25]. The alteration or protection of the intestinal mucosa by the gut microbiota is a key factor in the maintenance of the so-called gut barrier [26], which limits the access of microorganisms to the bloodstream and thus influences the inflammatory state described in processes such as obesity and MetS [27]. However, the action of the microbiota is not restricted to the gut, as its action extends to the central nervous system to influence food intake, via the gut–brain axis [28], and even to the liver to regulate nutrient metabolism, via the gut–liver axis [29]. This new scientific knowledge has made it possible to approach the treatment of metabolic diseases from a different angle, and offers a new therapy based on the modification of the microbiota through the use of probiotics [30].

## Methods

PubMed databases were used to search for reviews and research studies published in English using the search terms: sex steroids (testosterone and estrogen) and microbiota, sex steroids (testosterone and estrogen) and obesity, sex steroids (testosterone and estrogen) and metabolic syndrome, sex steroids (testosterone and estrogen) and diabetes, microbiota and gut barrier, microbiota and inflammation, microbiota and short chain fatty acids, microbiota and bile acids, microbiota and phytoestrogens, gut–brain axis. Publication dates were not limited in order to fully review the available literature. Following this search, an initial selection of articles was made according to their titles and abstracts. Subsequently, a second selection was made based on a critical reading of the articles.

## Interaction between gut microbiota and sex hormones

### Evidence for interaction between gut microbiota and sex hormones

**Fig. 1:**
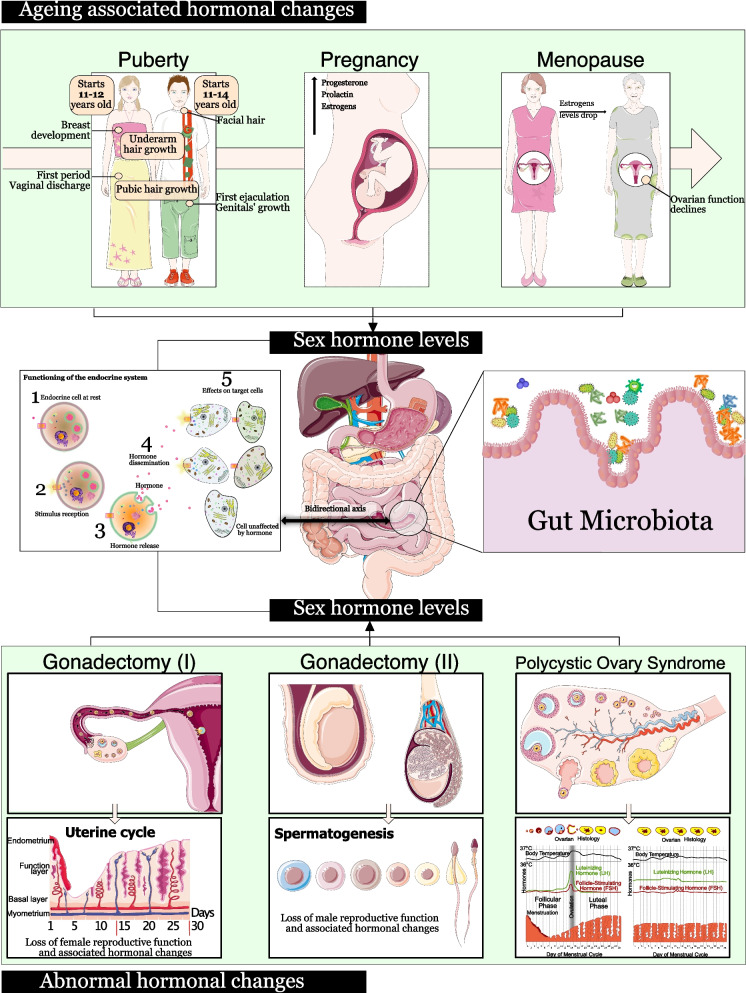
Interaction between gut microbiota and sex hormones. Various factors, such as puberty, pregnancy, menopause, polycystic ovary syndrome (PCOS), and gonadectomy, result in changes in sex hormone levels (testosterone and estradiol), which in turn lead to changes in the composition of the gut microbiota

The composition of the gut microbiota has been found to be sex-dependent [23] and may in turn influence sex hormone levels, influencing, for example, non-ovarian estrogen levels in men and postmenopausal women via the enterohepatic circulation (Fig. 1) [31].

#### Studies in rodents

Studies in mice have shown a change in estradiol and testosterone levels following microbial colonization [32, 33]. Regarding hormonal changes associated with puberty, no differences in microbial alpha-diversity have been observed in prepubertal mice, while the microbiota of post-pubertal mice shows a sex bias [34]. In this latter study, after reducing androgen levels by castration, the microbiota of castrated males showed more similarities with the microbiota of females than with the microbiota of gonadal-intact males.

Furthermore, gonadectomy has demonstrated the influence of sex hormones on the observed sexual bias in gut microbiota composition [35]. This study showed that testosterone treatment prevented the observed changes in gut microbiota composition in gonadectomized males. Along the same lines, we have described that gonadal hormone depletion in rats by gonadectomy, alone or combined with postnatal overfeeding, modified the gut microbiota towards a more deleterious profile, with a greater effect in females than in males, and mainly in the presence of an overfeeding condition [36]. In this study, we have identified several gut microRNAs (miRNAs) as potential mediators of the impact of changes in the gut microbiota on host physiology. We have also observed that exposure of female rats to high doses of androgens in early postnatal life not only persistently altered the sex steroid profile and several anthropometric and physiological parameters when subjected to obesogenic manipulations, but also impacted on the gut microbiota, with higher abundance of *Bacteroidetes* and lower *Firmicutes* in early adulthood, which disappeared after overfeeding in adulthood [37]. These changes in the microbiota were also related to the intestinal expression of several miRNAs. In view of the results presented here, it seems plausible that sex hormones may contribute to defining sex-dependent differences in the gut microbiota and that the interaction between microbiota and the host may be mediated by intestinal-derived miRNAs.

#### Human studies

Men and women with elevated serum testosterone and estradiol levels, respectively, harbored a more diverse gut microbiota, with a number of bacterial genera correlated with testosterone (*Acinetobacter*, *Dorea*, *Ruminococcus* and *Megamonas*) and estradiol (*Slackia* and *Butyricimonas*) levels [24]. In humans, it has been shown that the gut microbiota is influenced by changes in estrogen and androgen levels due to factors such as pregnancy, puberty, menopause, or PCOS. In this regard, women with PCOS (hyperandrogenic) show a markedly altered microbiota [38–40], as it changes from first to third trimester of pregnancy, with an overall increase in *Proteobacteria* and *Actinobacteria* and reduced richness [41].

Sex differences in gut microbiota composition increase at puberty, with girls' gut microbiota becoming more similar to that of adults with pubertal progression. These results might also suggest that gut microbiota may affect the timing of puberty, possibly by regulating host sex hormone levels [42–44].

In men and postmenopausal women, urinary estrogen levels have shown a strong association with gut microbiota richness and alpha-diversity, whereas premenopausal female estrogen levels, highly variable when collected during menstrual cycles, did not show this association [31, 45]. Recently, it has been reported that the gut microbiota of postmenopausal women is more similar to that of men than that of premenopausal women, with no significant differences actually observed between postmenopausal women and men of equivalent age [46, 47]. This study also showed an association between gonadal steroids and differences in microbiota, with steroid biosynthesis and degradation pathways being enriched in premenopausal women and significantly associated with plasma testosterone levels. In addition, the microbiota allowed prediction of circulating testosterone levels in both humans and (antibiotic-treated) male mice after transfer of human fecal material.

We have previously described in several studies a series of differences in the composition of the microbiota according to sex. In this regard, when studying the patterns of gut microbiota associated with obesity in men and postmenopausal women, according to sex and body mass index (BMI), we have observed a lower abundance of the genera *Bacteroides* (for a BMI over 33) and *Bilophila* in men, as well as a greater presence of the genera *Veillonella* and *Methanobrevibacter* [48]. In another study on differences in gut microbiota associated with sex and hormonal status conducted in premenopausal and postmenopausal women, together with their respective groups of control men, a higher proportion of *Firmicutes/Bacteroidetes* and the genera *Lachnospira* and *Roseburia* was observed in postmenopausal women, whose levels were similar to those of men. In contrast, the genera *Prevotella*, *Parabacteroides* and *Bilophila* showed lower levels in premenopausal women, whose levels were similar to those of men [47]. Another study on sex differences in the gut microbiota of patients with MetS showed a higher abundance of the genera *Collinsella*, *Alistipes*, *Anaerotruncus* and *Phascolarctobacterium*, as well as a lower abundance of the genera *Faecalibacterium* and *Prevotella* in women with MetS than in men with MetS [49]. Taken together, these results suggest that the sexual dimorphism observed in the incidence of metabolic diseases and their comorbidities might be, at least partially, related to differences in the composition of the gut microbiota between sexes and among women with different hormonal status.

### Mechanism of interaction between gut microbiota and sex hormones

#### Bile acids

It has recently been suggested that part of the sex bias of the gut microbiota may depend on bile acids, as the bile acid pool is larger in males than in females [50, 51]. After being synthesized in the liver from cholesterol, they are metabolized by the gut microbiota into secondary bile acids, which in turn can modify the structure of the microbiota and lead to various pathologies [52–54]. Thus, gut microbiota regulates the secondary metabolism of bile acids and inhibits their synthesis in the liver by regulating the expression of fibroblast growth factor 15 (FGF15) in the ileum and cholesterol 7α-hydroxylase (CYP7A1) in the liver through mechanisms dependent on the farnesoid X receptor (FXR) [55, 56], a nuclear receptor for bile acids. FGF15 represses the expression of CYP7A1 in the liver [57], the enzyme that catalyzes and regulates the first step of bile acid synthesis [58]. Furthermore, it has been observed that a reduction of bile acids leads to bacterial proliferation and that FXR inhibits bacterial overgrowth [59].

Several studies have confirmed the relationship between bile acids, sex hormones and the composition of the gut microbiota. In this way, administration of cholic acid to rats induced changes in the microbiota similar to those induced by high-fat diets, increasing levels of *Firmicutes* at the expense of *Bacteroidetes* [60]. In addition, transplantation of fecal microbiota (from a lean donor) produced changes in the gut microbiome and bile acid profiles similar to those of the lean donor [61], while gonadectomy in mice altered the bile acid pattern [35], as in germ-free (GF) and antibiotic-treated rats [62]. Since testosterone is synthesized from bile acids [63], and as described above, bile acid levels are altered by the microbiota, it is tenable that the microbiota might indirectly influence the level of testosterone.

#### Enzymatic action

The commensal microbial community can affect sex hormone levels through the activity of its enzymes. In this way, the term “strobolome” has been coined to define as the set of genes in the gut microbiota capable of activating estrogens from their inactive glucuronides, notably thanks to the enzymes β-glucuronidases, which deconjugate estrogens into their active forms [64–66]. These active estrogens pass into the bloodstream and act on estrogen receptors alpha (ERα) and beta (ERβ) [67]. Similarly, a recent study has concluded that the gut microbiota is involved in the metabolism and intestinal deglucuronidation of dihydrotestosterone (DHT) and testosterone, resulting in extremely high levels of the most potent androgen, DHT [68].

Another possible mechanism of action of the gut microbiota in sex bias could be found in its hydroxysteroid dehydrogenase (HSD) enzymes, which are involved in the metabolism of steroid hormones and control the binding of steroids to their nuclear receptors, making them act as activators or inhibitors [69, 70].

#### Phytoestrogens

In addition to the three main forms of estrogens (cholesterol-derived steroid hormones), estradiol (E2, predominant in non-pregnant women before menopause), estrone (E1, predominant after menopause) and estriol (E3, predominant during pregnancy), there are plant compounds, called phytoestrogens, which show structural and functional similarities to estrogens [71]. Phytoestrogens include isoflavones, such as genistein and daidzein, which are mainly abundant in soya and are activated after being metabolized by the gut microbiota [72]. In this sense, the intestinal microbiota allows O-desmethylangolensin (ODMA) and equol to be obtained from daidzein, both of which have estrogenic activity [73–76].

Similar to estrogens, phytoestrogens cause physiological effects by affecting cell signaling, as they can induce or inhibit estrogen action by activating or inhibiting ERα or ERβ, and may trigger also epigenetic effects and intracellular signaling cascades [77–79]. Related to this, several human studies suggest that phytoestrogens can ameliorate various pathologies by modulating the endocrine system, including menopausal symptoms [72], and can reverse symptoms of metabolic endotoxemia [80]. In this regard, the phytoestrogen metabolite, equol, has been associated with a reduced risk of female hormone-related diseases by promoting urinary excretion of estrogen and modifying its blood levels in women [81, 82], while non-production of ODMA has been associated with obesity [73, 74].

Phytoestrogens are consumed in the diet, as they appear in fruits, veggies, legumes, and some grains. Indeed, dietary composition has an acute effect on the gut microbiota ecosystem [83]. A plant-based diet appears to be beneficial for human health by promoting the development of more diverse and stable microbial systems [84]. From the three basics bacterial enterotypes [85], the one rich in *Prevotella* is associated to those individuals who consume less animal products and more plant-based foods [84]. In contrast, the enterotype rich in *Bacteroides* has been positively correlated with consumption of diets rich in animal protein and saturated fat. This is likely due to their ability to tolerate bile, which is common in the intestinal environments of those who consume animal products [86, 87]. Finally, the third enterotype is the one rich in *Ruminococcus*, whose biological significance is less evident [88].

## Key aspects of gut microbiota action in metabolic diseases

**Fig. 2:**
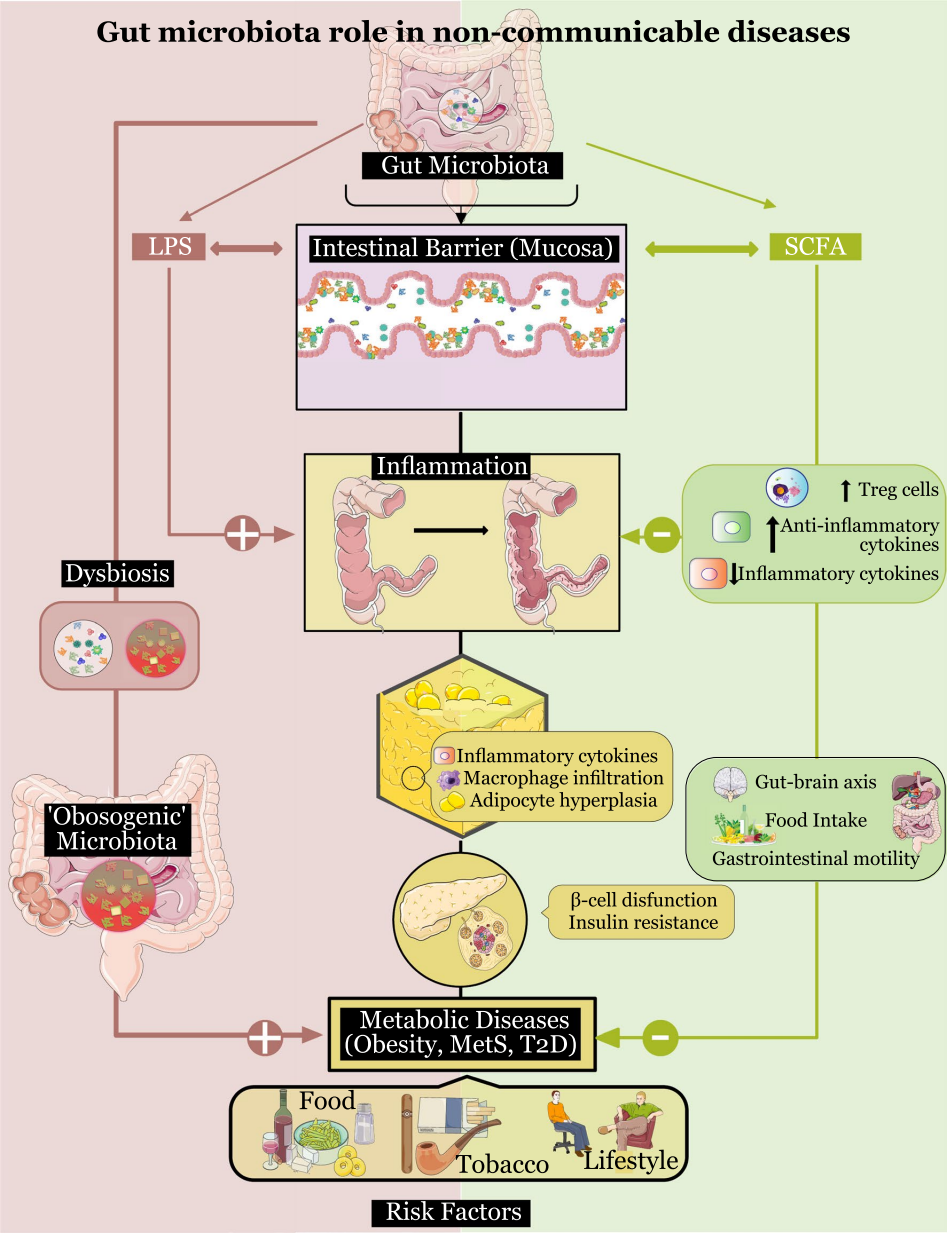
Involvement of gut microbiota in metabolic diseases. An "obesogenic" microbiota (higher *Firmicutes*/*Bacteroidetes* ratio), with a greater capacity to extract energy from the diet, may contribute to the state of obesity. Metabolic diseases are associated with chronic low-grade inflammation and the resulting imbalances in adipose tissue and pancreas. The microbiota can influence the inflammatory state via lipopolysaccharide (LPS), the gut barrier, and several of its metabolites (especially short-chain fatty acids (SCFAs)). LPS potentiates inflammation by inducing macrophage infiltration and pro-inflammatory cytokines in adipose tissue. The structure and permeability of the intestinal barrier (mucosa), which protects against inflammation by preventing bacterial translocation, is affected, positively or negatively, by the presence or absence of different types of bacteria. SCFAs improve the intestinal barrier by reinforcing tight junctions, reduce inflammation by increasing regulatory T cells (Treg cells) and anti-inflammatory cytokines and decreasing inflammatory cytokines, and improve glucose homeostasis and insulin sensitivity. SCFAs also intervene in the gut–brain axis by regulating the levels of hormones involved in the control of gastrointestinal motor function and food intake, such as leptin, ghrelin, peptide tyrosine tyrosine (PYY), cholecystokinin (CCK), and glucagon-like peptide-1 (GLP-1). *MetS* metabolic syndrome, *T2D* type 2 diabetes

Since the discovery in 2005 of an increased *Firmicutes/Bacteroidetes* ratio in obese compared to lean mice [89], many studies have addressed the role of the gut microbiota in obesity and associated pathologies, such as MetS and T2D [90]. The putative mechanisms whereby the microbiota contribute to these processes lies especially in the actions of lipopolysaccharide (LPS), the maintenance of the intestinal barrier, the by-products of its metabolism, and its intervention in the gut–brain axis (Fig. 2).

### Inflammation

Gut microbiota has been linked to diseases characterized by chronic low-level inflammation, such as obesity and T2D. Specifically, the inflammatory state is mainly influenced by LPS, the intestinal barrier, and several metabolites derived from bacterial metabolism.

#### Lipopolysaccharide

The LPS, an endotoxin from the outer membrane of Gram-negative bacteria, is involved in chronic low-grade inflammation by inducing the secretion of potentially diabetogenic pro-inflammatory cytokines and key components of the innate immune response in adipose tissue [91]. In addition, a high concentration of LPS in the bloodstream, defined as metabolic endotoxemia, has been linked to insulin resistance, adipocyte hyperplasia and reduced pancreatic beta-cell function [92]. Related to this, the genus *Prevotella*, which is in principle beneficial by producing short-chain fatty acids (SCFAs) [93], using a wide variety of polysaccharides [94], has also been described as detrimental by inducing tumor necrosis factor alpha (TNF-α) production by a LPS-induced mechanism [95] and producing phosphorylated dihydroceramide lipids, which in turn lead to the secretion of pro-inflammatory cytokines, as IL-6 [96].

The link between elevated levels of circulating LPS and metabolic diseases has been proven by chronic infusion of LPS in mice, which resulted in increased fasting blood glucose, hyper-insulinemia, and insulin resistance, as well as increased macrophage infiltration in adipose tissue [97]. In addition, the above study showed that ablation of the LPS co-receptor, CD14, reversed LPS-induced metabolic diseases.

Two non-exclusive mechanisms of LPS absorption from the gut into the circulatory system have been proposed [98]: (1) chylomicron-facilitated transport (lipoproteins that transport dietary lipids to peripheral tissues), supported by the fact that LPS secretion increases when cells are stimulated with fatty acids that promote chylomicron formation, while inhibition of chylomicron formation blocks LPS uptake; and (2) extracellular transport through the epithelial tight junctions, supported by the fact that reducing intestinal permeability and improving tight junction integrity reduces plasma LPS levels, circulating inflammatory cytokine concentrations, and hepatic inflammation.

#### Gut barrier integrity

The small intestine has an unattached mucus layer, while the colon has two layers, the inner, attached layer, and the outer, less dense and unattached layer [99]. The mucus layer of the intestinal epithelium, which is composed of glycans, or mucins (highly glycosylated proteins secreted by goblet cells, most notably the MUC2 protein), and forms the so-called intestinal barrier, represents a barrier to intestinal bacteria, providing protection against inflammation [27] involved in the pathogenesis of insulin resistance, which in turn is linked to obesity and T2D [100]. In this regard, the gut microbiota is known to influence the integrity and permeability of the intestinal barrier and thus the inflammatory state, due to its interaction with mucin-type O-glycans [25, 26], which in turn may lead to the development of metabolic diseases, such as insulin resistance. Related to this, increased intestinal permeability has been associated with T2D risk [101], and the low-grade inflammation and insulin resistance that characterize both obesity and T2D are mediated by bacterial LPS (metabolic endotoxemia) [25, 97]. Indeed, in GF mice the presence of gut microbiota is necessary for the maintenance of the gut mucosal structure [102, 103] and this structure can be modified by the transfer of fecal microbiota [104].

*Akkermansia muciniphila* and representatives of the genera *Bifidobacterium* and *Lactobacillus* are among the bacterial species that improve intestinal barrier integrity and inflammation, which is why they have long been used as probiotics [30]. In general, probiotic administration leads to an improvement in several factors related to obesity and MetS, such as increased intestinal permeability, and therefore a reduction in LPS translocation and low-grade systemic inflammation, while also improving hypothalamic insulin resistance and glucose tolerance [105, 106].

Specifically, *Akkermansia muciniphila* is able to degrade mucin [107], and plays a prominent role in intestinal barrier integrity and inflammatory processes. In terms of intestinal barrier integrity, this bacterium is able to restore the thickness of the mucous layer by increasing mucin-producing goblet cells [108, 109] and restore its integrity by inducing intestinal expression of occludin (intercellular junction proteins) [110, 111]. Moreover, the Amuc_1100 protein, specific to the outer membrane of this bacterium, improves the intestinal barrier and various processes of intestinal physiology by interacting with Toll-like receptors (TLR) 2 and 4 [112–114], while inducing the production of the anti-inflammatory cytokine, IL-10. This bacterium also contributes to the decrease in adipose tissue inflammation by reducing macrophage infiltration, restoring regulatory T cells (Treg cells), reducing pro-inflammatory cytokines (such as IL 6 and IL-1β), and increasing anti-inflammatory factors (such as α-tocopherol and β-sitosterol) [109, 111, 115].

Different species of *Lactobacillus* genus are able to ameliorate damage to the intestinal barrier caused by other bacteria [116]. *L. plantarum* is widely cited as enhancing intestinal barrier integrity by improving epithelial tight junctions [117–119], while inhibiting the inflammatory response by reducing the expression of pro-inflammatory cytokines through modulation of TLR, nuclear factor kappa B (NF-κB) and mitogen-activated protein kinase (MAPK) signaling pathways [120, 121], and inducing the secretion of human β-defensin 2, a peptide involved in host defense [122]. Similar effects have also been observed with *L. fructosus*, *L. acidophilus*, *L. fermentum*, *L. casei* and *L. rhamnosus* [123–129].

*Bifidobacterium* genus improves the intestinal barrier by increasing tight junction proteins [130] and modulating goblet cell function by secreting metabolites, thereby increasing the production of MUC2 [131]. In addition, this genus also induces an increase in intestinal Reg I proteins [132], which play a prominent role in the villous structure of the small intestine [133]. Moreover, these bacteria reduce inflammation by several mechanisms: (1) decreasing pro-inflammatory cytokines (IL-6 and IL-17) and increasing anti-inflammatory cytokines (IL-4 and IL-10) [130, 134]; (2) decreasing bacterial translocation [135–137]; (3) preventing LPS uptake into the bloodstream [130]; and (4) enhancing macrophage and dendritic cell function in relation to phagocytosis, cytokine production and induction of T-lymphocyte proliferation [134].

#### Gut microbiota-derived metabolites

SCFAs (mainly acetic, propionic and butyric acids) from bacterial fermentation of dietary fiber have been linked to a decrease in inflammation [138, 139], as well as improved glucose homeostasis and insulin sensitivity [140]. These compounds improve gut barrier function and inflammatory status through several mechanisms: (1) upregulation of intestinal tight junction proteins [141–143]; (2) regulation of tight junction assembly via an activation-dependent mechanism of AMP-activated protein kinase (AMPK) [144–146]; (3) increase in Treg cells [141, 147]; and (4) increase in anti-inflammatory cytokines and decrease in inflammatory cytokines [147, 148].

The inflammatory state is highly dependent on the balance between Treg cells producing the anti-inflammatory cytokine, IL-10, and T-helper (Th) 17 cells producing the inflammatory cytokine, IL-17, so that an increase in the Treg/Th17 ratio reduces the inflammatory state. In this sense, treatment of inflammatory bowel disease with parthenolide (a sesquiterpene lactone originally extracted from the shoots of the plant, *Tanacetum balsamita*) reduces inflammation in a gut microbiota-dependent manner, as it improves the Treg/Th17 balance in the intestinal mucosa through increased production of SCFAs [149]. Related to this, butyrate plays a key role in regulating the Treg/Th17 balance by inducing intestinal Treg cells differentiation in a histone acetylation-dependent mechanism in the promoter regions of certain genes, via inhibition of histone deacetylase [150, 151]. This increase in Treg cells translates into increased levels of anti-Th17 cytokines (IL-10 and IL-12) and reduced levels of IL-17 [152].

In addition to the increase of the anti-inflammatory cytokine IL-10 and the reduction of the pro-inflammatory cytokine IL-17 cited above, SCFAs also appear to be involved in the reduction of other pro-inflammatory factors, such as TNF-α, IL-1β, IL-6, and NO [153, 154], and in the inhibition of NF-κB activity [155, 156], which has been linked to inflammatory processes [157].

In addition, bacterial metabolites other than SCFAs, such as 4-hydroxyphenylpropionic acid, 4-hydroxyphenylacetic acid and caffeic acid, may mediate inflammation, possibly by mediating the aryl hydrocarbon receptor (AHR) and modulating the Treg/Th17 ratio [158]. In line with this, secondary bile acids resulting from bacterial deconjugation of bile acids have been reported to enhance Treg cells differentiation in the gut [159, 160].

### Gut–brain axis

It is widely reported that the influence of microbiota on the development of obesity and related pathologies may be due in part to altered levels of intestinal hormones involved in the gut–brain axis, so that the central nervous system regulates food intake through the products of gut microbiota activity, including SCFAs [28]. Interestingly, the absence of gut microbiota may induce the consumption of obesogenic nutrients, such as fats and sugars, due to increased expression of their receptors [161, 162]. Together, these latter compounds appear to mediate the control of gastrointestinal motor function and food intake [163, 164]. Interestingly, the gut–brain axis activated by GLP-1 for the control of insulin secretion and gastric emptying has been reported to be affected by a set of ileum bacteria [165]. More specifically, microbially derived SCFAs have been found to induce an increase in GLP-1 levels [127, 166, 167]. Conversely, both peptide tyrosine tyrosine (PYY) and cholecystokinin (CCK), produced by intestinal L-cells, are considered anorectic hormones that inhibit food intake and reduce weight gain [168, 169].

GLP-1 is an incretin hormone whose action on insulin release from pancreatic b-cells to maintain normoglycemia has been described [170, 171]. Moreover, GLP-1 reduces the entry of nutrients into the circulation by increasing satiety and reducing the rate of gastric emptying [172, 173]. More specifically, GLP-1 has been shown to modulate central mechanisms of food intake in the hypothalamus by stimulating the activity of proopiomelanocortin (POMC) anorexigenic neurons and inhibiting the activity of agouti-related protein (AgRP)/neuropeptide Y (NpY) orexigenic neurons [174].

Both the orexigenic hormone, ghrelin, and the anorexigenic hormone, leptin, play a key role via the gut–brain axis in metabolic regulation and energy homeostasis and thus in the development of obesity [163, 175, 176]. Ghrelin is linked to adiposity and excessive weight gain by inducing an increase in gastric emptying rate and a decrease in energy expenditure [177–179], while increasing food intake by stimulating orexigenic AgRP/NpY neurons and inhibiting anorexigenic POMC neurons in the hypothalamus [180]. It is important to note that ghrelin is also involved in GH secretion [181, 182], which plays a key role in sexually dimorphic gene expression. In this sense, the sexual dimorphism observed in metabolic diseases could be due, at least in part, to the influence of the microbiota on ghrelin levels and thus on GH release. Regarding leptin, it is known to reduce food intake, body weight and circulating insulin, elevate circulating concentrations of n-octanoyl ghrelin, and promote the release of GH [183–185].

As an additional component of this gut–brain axis, there is solid evidence for the impact of conditions of stress and chronic activation of the hypothalamic–pituitary–adrenal (HPA) axis on the composition of the gut microbiota, as well as on intestinal permeability [186]. Considering that glucocorticoid stress responses are sexually distinct [187], this might represent an additional mechanism for sex divergences in gut microbiota composition, and its influence on metabolic health. Furthermore, since chronic activation of the HPA axis is linked to suppression of gonadal function [188], stress may also indirectly alter the microbiome by inhibiting sex steroid levels in both sexes. However, the actual contribution of this adrenal pathway to setting the physiological sex differences in gut microbiota and, thereby, in metabolic disease remains largely unexplored.

## Obesity

Obesity, established for a BMI of 30 kg/m^2^ or higher, has increased in prevalence in the developed world in both adults and children. This pathology, which is the result of complex genetic, socio-economic and cultural relationships, leads to serious health, economic, and social problems [1]. Scientific evidence has shown that the development of some metabolic disorders is related to the distribution of body fat, and that this distribution shows sexual dimorphism. In this sense, fat tends to accumulate around the trunk and abdomen in men (android distribution) and around the hips and thighs in women (gynoid distribution) [5]. Abdominal adiposity, and especially visceral adiposity, has been associated with increased metabolic complications in both men and women [8, 189, 190] by causing an increase in blood glucose and triglycerides, a decrease in high-density lipoproteins (HDL) cholesterol and an increase in low-density lipoproteins (LDL) particles, as well as an increase in inflammatory markers [191]. On the contrary, gluteo-femoral fat is associated with a protective lipid and glucose profile and decreased metabolic risk, appearing to exert its protective effect through long-term fatty acid storage and a beneficial adipokine profile (positive association with leptin and adiponectin levels and negative association with the level of inflammatory cytokines) [9] (Table 1).

**Table 1:** Summary of the influence of elevated (↑) and decreased (↓) values of the sex hormones testosterone (T) and estradiol (E) on obesity, metabolic syndrome (MetS), and type 2 diabetes (T2D)

	Men	Women
↑T	Reduction of central obesity Decrease in visceral fat	Increase in central obesity Increase in MetS Increase in T2D
↓T	Increased fat mass (subcutaneous fat accumulation, not intra-abdominal fat accumulation) Increased adiposity (preferential accumulation of visceral abdominal fat) (ageing) Increased MetS Increased T2D	
↑E		Increased T2D (non-physiological value)^a^
↓E		Increase in central obesity Increased MetS Increase in T2D (non-physiological value)^a^

^a^A non-physiological value of estradiol (increased or decreased) would be responsible for the same effect, the increased risk of developing T2D

### Role of sex hormones in obesity

A body of evidence supports the view that sex steroids modulate body fat distribution. In this regard, pubertal hormonal changes have been associated with different body weight gain between the sexes, due to increased lean mass in boys and increased fat mass in girls, and with android and gynoid fat distribution [6]. Furthermore, several studies have shown the involvement of some genes in the sexual dimorphism observed in body fat distribution, as well as the potential role of sex steroid hormones in the regulation of these genes [7, 192, 193].

In men, testosterone inhibits the uptake of triglycerides in the intra-abdominal region and appears to promote their accumulation in the subcutaneous region [194], while causing a reduction in catecholamine-stimulated lipolysis in subcutaneous but not in visceral fat [195]. These processes appear to be influenced by the androgen receptor (AR) gene, as in AR knockout mouse models, deletion of the AR causes an increase in adiposity, and especially late adiposity, by decreasing lipolysis [196, 197]. Furthermore, it appears that protein caveolin-1 (CAV1) plays an important role in fat accumulation and that it is regulated differently by estrogens (estradiol) and androgens (DHT) [198].

At the cellular level, differences in the effect of sex steroids (androgens and estrogens) on adipocyte function in white adipose tissue have been observed, regarding key aspects such as adipocyte differentiation, lipolysis/lipogenesis, insulin sensitivity, and adipokine production/secretion [199]. In this context, testosterone and DHT regulate the differentiation of pluripotent mouse mesenchymal cells, promoting and inhibiting their differentiation into myocytes and adipocytes, respectively, in an AR-dependent manner [200]. Similarly, in an in vitro study with human cells, DHT inhibited adipogenic differentiation of human mesenchymal stem cells and human preadipocytes in an AR-dependent manner, increased lipolysis and reduced lipid accumulation [201]. In castrated mice (a model of male hypogonadism), fat mass increased through adipocyte hypertrophy and adipogenesis [202], whereas when these mice were subjected to hormone replacement therapy, testosterone prevented the expansion of visceral and subcutaneous fat mass. In addition, obesogenic adipogenesis was also elevated by inhibiting AR activity. This study also showed differential regulation of fat distribution, with testosterone-derived estradiol and DHT blocking the increase in visceral and subcutaneous fat, respectively.

At the enzymatic level, the action of lipoprotein lipase (LPL), a key enzyme in lipid uptake and storage by adipocytes [203], appears to be suppressed by estradiol in the adipose tissue of obese women [204] and by testosterone in the adipose tissue of obese men [205], with this suppression being greater in the thigh than in the abdomen in the case of men, and could therefore be a key element in their central fat accumulation. Furthermore, testosterone deficiency in men increases LPL and acyl-CoA synthetase (ACS) activity and induces fatty acid accumulation in femoral adipose tissue [206, 207], and testosterone replacement reduces abdominal LPL activity and triglyceride uptake in this area [208]. As for the influence of female steroids, in women, sex steroid deficiency after menopause influences ACS and diacylglycerol acyltransferase (DGAT) activity and promotes increased storage of fatty acids in subcutaneous adipose tissue [209]. In addition, in premenopausal women, femoral adipogenic factors respond to acute sex hormone suppression to a greater extent than abdominal ones, and estrogen and progesterone appear to have different effects on the regulation of fatty acid metabolism [210].

### Obesity in men

Testosterone concentrations have been negatively correlated with central obesity [211, 212], and testosterone treatment has been found to decrease visceral fat in men with symptoms of androgen deficiency and low-normal serum testosterone levels [17]. In this context, oxandrolone, an artificial steroid similar to testosterone, reduced total, abdominal and peripheral fat, but mainly total and abdominal fat, in elderly men [213]. In this study, visceral adipose tissue decreased to a greater extent than subcutaneous adipose tissue in the abdominal region. In addition, testosterone replacement therapy improved glycemic control, insulin resistance, and dyslipidemia in patients with hypogonadism, partly by reducing central obesity [18, 214, 215]. On the other hand, androgen deprivation therapy in men with prostate cancer leads to an increase in fat mass [14, 216, 217]. In relation to this, and contrary to what might be assumed, it has been described that the increase in abdominal fat is due to the accumulation of subcutaneous fat rather than intra-abdominal fat [218, 219]. Furthermore, the decline in testosterone with aging is accompanied by increased adiposity, with a preferential accumulation of abdominal fat and a greater accumulation of visceral adipose tissue [220]. It has also been reported that visceral adipose tissue correlates inversely with bioavailable and free testosterone, and that subcutaneous adipose tissue correlates negatively with sex hormone binding globulin (SHBG) [221]. A more recent study in male twins has shown an inverse correlation between the amount of subcutaneous fat and serum concentrations of total and free testosterone, DHT and SHBG, as well as between intra-abdominal fat and total testosterone and SHBG [222]. However, it has also been pointed out that low testosterone concentration might be linked with an increase in total body fat rather than with an excess of visceral fat; observations that underline the importance of adrenal steroids in body composition in men [223]. Finally, fat redistribution after sex steroid treatment is also observed in transsexual men [10, 224, 225].

### Obesity in women

In women, central obesity has been correlated with increased testosterone levels and decreased estradiol [211]. The hormonal changes of menopause lead to a redistribution of fat, independent of total fat and age, towards a more central and android phenotype [11, 226, 227]; yet, some studies have suggested that the distribution of upper body fat after menopause may be due to ageing rather than menopause per se [228, 229]. Recently, body or trunk fat mass has been associated with lower total estradiol and higher calculated free estradiol concentrations in premenopausal women, as well as higher concentrations of total and calculated free testosterone and lower concentrations of SHBG and insulin-like growth factor-I (IGF-I) in both premenopausal and postmenopausal women [230]. Related to this, the shift towards central and android fat distribution observed in perimenopausal and postmenopausal women may be counteracted by hormone replacement therapy [231]. In addition, women with hyperandrogenism due to PCOS show increased central adiposity [20, 232, 233]. Also remarkably, fat redistribution is observed in transgender women after sex steroid treatment [10, 224, 225].

## Metabolic syndrome

MetS is a pathological condition characterized by abdominal obesity, insulin resistance, hypertension and hyperlipidemia, which has spread across the globe and contributes to the rising prevalence of diseases, such as T2D, coronary heart disease, and stroke [3] (Table 1).

### Role of sex hormones in metabolic syndrome

There is a large body of scientific evidence confirming the role of sex hormones in the development of MetS. An inverse association between serum SHBG levels and the prevalence of MetS has been observed in children aged 12–16 years, with SHBG being a more sensitive marker of MetS in boys, but not in girls, indicating sexual dimorphism already at an early age [234]. At older ages, an association between lower SHBG levels and MetS is still observed in both males and females, while total and free testosterone levels are lower in males and higher in females with MetS [235–237]. However, it has been suggested that low SHBG level would be associated with a higher prevalence of MetS in both men and premenopausal women, but not in postmenopausal women, so that plasma SHBG level could be a significant predictor of MetS only in men and women before menopause [238].

The sexual dimorphism observed in the influence of testosterone on MetS appears to be AR-dependent, and several mechanisms have been suggested to explain the association between testosterone level and MetS [239]. In men, there is evidence of an inverse correlation between testosterone and the development of visceral obesity, insulin resistance and MetS [240, 241]. Along these lines, the AR-mediated anti-obesity effect of testosterone has been reported in both men [242] and rodents [196, 243]. In women, elevated testosterone levels have been reported to be associated with insulin resistance and glucose intolerance by decreasing whole-body glucose uptake [244–246]. Regarding the action of testosterone on the pancreas, a study in mice has shown that the AR regulates male pancreatic beta-cell physiology, so that a deficiency of this receptor decreases glucose-stimulated insulin secretion and leads to glucose intolerance [247]. Conversely, it has been proposed that an excess testosterone could lead to pancreatic beta-cell dysfunction in women by an AR-dependent mechanism [248], with impaired insulin secretion [249].

At the central nervous system level, studies in rodents have confirmed that AR expression is higher in the brains of males than in females, where this receptor favors the central action of leptin [250]. Another study has shown that androgen excess in female mice prevents the activation of brown adipose tissue thermogenesis by leptin, which is associated with lower energy expenditure and visceral obesity, while hypothalamic expression of POMC decreases [251], suggesting that the increase in visceral adiposity in hyperandrogenic women may have a central origin.

### Metabolic syndrome in men

In men, MetS appears to be related to testosterone, but not to estradiol [252, 253]. In this regard, testosterone levels have been negatively associated with MetS risk [254], while testosterone replacement therapy appears to improve most MetS parameters (glycemia, triglyceride levels, waist circumference, and high-density lipoprotein cholesterol) [255]. In addition, a recent study has shown that the negative association between testosterone and MetS holds true for all MetS components [256].

Moreover, several articles specify that MetS is inversely associated with both total testosterone and SHBG [257–259], and that both testosterone and SHBG show an inverse association with insulin, glucose and triglyceride concentrations, as well as a positive association with HDL cholesterol [260–262]. Moreover, numerous articles point to SHBG levels as the most influential in the development of MetS [263, 264] and as an independent and dominant risk factor [265–267] and a good early marker of MetS [257, 258].

As for free testosterone, although its inverse association with MetS has also been reported, most articles indicate that this association is smaller than in the case of total testosterone and SHBG [268–270], and it has even been reported that this relationship does not exist [267] or that it may be positive [264].

In relation to the above, men with hypogonadism (testosterone deficiency), resulting from androgen deprivation therapy for prostate cancer, show lower levels of total and free testosterone, as well as a higher prevalence of MetS [15]. Within the MetS parameters, these men had a higher prevalence of abdominal obesity and hyperglycemia, as well as elevated triglyceride levels compared to controls. In line with this, testosterone treatment in men with hypogonadism restores physiological testosterone levels and improves MetS components, increasing HDL and reducing total cholesterol, LDL cholesterol, triglycerides, and glucose [18, 214, 271].

### Metabolic syndrome in women

The level of estrogen also appears to influence the prevalence of MetS. Thus, oophorectomy-induced estrogen depletion in rats induces a worsening of most MetS components (lipids, glucose, HDL, and LDL) [272, 273], while in women under 50 years of age, i.e., undergoing menopause, its prevalence increases [274, 275]. Furthermore, in women who have suffered hysterectomy (often accompanied by bilateral oophorectomy to prevent subsequent ovarian cancer) an increase in blood glucose level [276] and hypertension [277] has been reported.

Menopause causes a decrease in the level of SHBG, at least partially due to a decrease in estrogen, while the level of testosterone is not altered during the menopausal years [278]. In this sense, menopause can be considered a predictor (risk factor) of MetS and all its individual components independent of age [279, 280]. Furthermore, an inverse association between SHBG and MetS has been described, especially among postmenopausal women [281].

As for testosterone, its excess (hyperandrogenism) in women with PCOS is a powerful predictor of the metabolic disorders characteristic of MetS, with this pathology being more prevalent in patients with PCOS than in healthy women [21, 282]. However, although the scientific literature widely gives hyperandrogenism a prominent role in the metabolic disturbances associated with PCOS [283], a recent review and meta-analysis study has shown that the higher prevalence of MetS in women with PCOS is associated with obesity and metabolic characteristics, but not with the hyperandrogenism index [284].

## Type 2 diabetes

The term diabetes encompasses a group of diseases, differentiated by their mechanisms of development, that reduce the ability to regulate the level of glucose in the blood stream and lead to prolonged hyperglycemia. There are two primary forms of diabetes, insulin-dependent diabetes (type 1 diabetes, T1D) and non-insulin-dependent diabetes (type 2 diabetes, T2D), due to autoimmune and metabolic processes, respectively. T2D is characterized by insufficient insulin production by pancreatic b-cells and impaired hepatic glucose metabolism, as well as insulin resistance, leading to reduced tissue responsiveness to insulin [285, 286]. The emergence of this pathology, which has become a pandemic, affecting approximately 9% of the world's population [2], is conditioned by several factors, such as genetics, sedentary lifestyle, physical inactivity, smoking, alcohol consumption, oxidative stress, and diet [287]. However, obesity is considered to be the major risk factor for T2D, which influences both the development of insulin resistance and the course of the disease [288]. In the present review, we have considered only T2D because of its metabolic disease character (Table 1).

### Role of sex hormones in diabetes

Impaired fasting glucose (IFG) and impaired glucose tolerance (IGT), which occur as a preliminary step to T2D, show sexual dimorphism, with IGT being more frequent in women and IFG in men [289–291]. It has been suggested that sex hormones may be responsible for this dimorphism. Indeed, estrogen treatment of menopause lowers fasting glucose and worsens glucose tolerance [290]. Moreover, it has been confirmed that the incidence of T2D is higher in men than in women [292, 293], which further supports the involvement of sex hormones in the development of this pathology. In addition, menopause implies an increased risk of T2D, whereas hormone therapy for menopause may delay the onset of T2D [12].

### Diabetes in men

Men with T2D have lower levels of total testosterone and free testosterone [294–296]. Related to this, it has been suggested that low levels of testosterone and SHBG are linked to the development of insulin resistance and subsequent T2D in men [8, 254]. In addition, the combination of high levels of SHBG and low levels of testosterone has been associated with increased mortality in men with T2D [297, 298]. Furthermore, other studies have shown that in men with T2D, low testosterone levels per se are associated with increased mortality, whereas testosterone replacement may improve survival in these men [299, 300]. In the same way, it has been reported that the proportion of men with T2D was reduced after 2 years of testosterone treatment [18, 301]. In addition, androgen deprivation therapy in prostate cancer has been found to induce an increased risk of diabetes [16, 302, 303].

In line with the above, men with T2D tend to have low testosterone levels, and most of them have hypogonadism [304]. Indeed, numerous studies have confirmed that obese T2D patients with hypogonadism and low testosterone levels show improved insulin resistance and glycemic control after undergoing testosterone replacement therapy (TRT) [18, 271, 305].

With regard to female hormones, men with high estradiol levels have an increased risk of T2D, and this high estradiol concentration, together with a low SHBH concentration, carries an additive detrimental effect on the risk of T2D in men [8, 306].

### Diabetes in women

In contrast to men, high testosterone levels in women are linked to insulin resistance and T2D [254, 307, 308]. However, one study has shown that although elevated SHBG values in Chinese women are associated with a lower likelihood of T2D, estradiol and testosterone levels show no association with T2D in this ethnic group [306]. These contradictory results regarding the relationship between testosterone and the incidence of T2D may be due to the measurement of testosterone, with some authors using total testosterone and others using free testosterone, and according to a recent study, the method of analysis may differ between studies [309]. In addition, the free androgen index (FAI) is not a reliable indicator of free testosterone when the SHBG concentration is below 30 nmol/L, which would lead to possible research errors in women with low SHBG levels [310]. Accordingly, it has been reported that in women there is no association between total testosterone and T2D, although a higher level of free testosterone is associated with an increased risk of T2D [311].

As in men, the level of SHBG has also been inversely associated with the risk of T2D in women [254, 281, 295, 312]. In fact, in women, the association between low SHBG and T2D appears to be stronger than in men [307, 308]. Although this inverse association between SHBG and T2D is persistent in different ethnic groups [313], according to a study in postmenopausal Hispanic women with and without T2D, mean SHBG levels were not significantly different in the two groups [314]. These contradictory results may be due to the fact that sex hormone and SHBG levels may vary in postmenopausal women according to racial/ethnic differences [315, 316].

With respect to estradiol, postmenopausal women with T2D have been reported to have higher estradiol levels than healthy women [307, 312, 314]. However, data from a body of evidence based on earlier menarche or menopause and the practice of hysterectomy and oophorectomy suggest that non-physiological estradiol levels (higher or lower than normal values) may be responsible for an increased incidence of T2D. In this respect, early onset of menarche appears to increase the risk of T2D [317–319]. Nevertheless, some studies suggest that part of the risk of T2D due to early menarche may be due to the increased adiposity [22, 320, 321], as early menarche has been shown to be associated also with an increase in BMI in adulthood [322, 323]. On the other hand, early menopause or premature ovarian insufficiency leads to an increased risk of developing T2D [324–326]. Similar results have been observed in postmenopausal women with bilateral oophorectomy [22, 327]. Finally, hysterectomy accompanied by bilateral salpingo-oophorectomy (BSO) showed a higher risk of T2D than hysterectomy per se [327]. However, other studies have associated hysterectomy with an increased risk of T2D, while BSO per se or together with hysterectomy did not increase the risk of T2D [328, 329]. Pandeya et al. indicated that women who underwent hysterectomy or oophorectomy show an increased risk of developing T2D, but does not differentiate whether the two conditions occurred separately or together [22]. Another study showed that, relative to intact women, hysterectomized women with bilateral oophorectomy had lower levels of both total and bioavailable testosterone, while hysterectomized women with ovarian preservation had intermediate levels [330]. This study also revealed that hysterectomized women with bilateral oophorectomy tended to have lower total estradiol levels, while bioavailable estradiol and SHBG levels did not differ between hysterectomy and oophorectomy status. Related to this, hormone therapy with estrogen and progestin in postmenopausal women (both with intact uterus and hysterectomized) reduced the incidence of diabetes [13, 331, 332].

## Perspectives and significance

In this review, we focused the role of sexual hormones in the development and prevalence of metabolic diseases such as obesity, metabolic syndrome and type 2 diabetes. Sex steroids, mainly estrogens and testosterone, are implicated in the sexual dimorphism in the structure and composition of the gut microbiota. Taking into account this relationship, it is plausible the contribution of their interconnections in the development of disease, and the subsequent differences between sexes. This influence is reflected both between men and women, and among women themselves due to hormonal changes associated with the menopause. The mutual interaction between sex steroids and the gut microbiota plays a prominent role in the development of metabolic diseases, highlighting the role of the microbiota in key aspects, such as gut barrier integrity, inflammatory status and the gut–brain axis.

The relevance of this field lies in the fact that fecal transfer and modification of the composition of the microbiota with specific diets, prebiotics, probiotics or synbiotics has attracted considerable interest in recent years as a potential alternative therapeutic tool for the treatment of metabolic diseases. In fact, the intestinal microbiome is currently considered an important therapeutic target, since specific changes in the bacterial community could help alleviate associated metabolic diseases.

Moreover, the identification of the mechanisms responsible for sexual dimorphism in the incidence of metabolic diseases has special importance when developing effective strategies and therapies aimed at reducing their incidence. The composition of the gut microbiota depends on the interaction with sex hormones in addition to other factors, such as the nutritional habits of the host organism, so the therapies to treat the dysbiosis of the gut microbiota associated with these diseases may have sex-specific effects.

## Data Availability

Not applicable.

## Abbreviations

AgRP: Agouti-related protein
ACS: Acyl-CoA synthetase
AR: Androgen receptor
BMI: Body mass index
BSO: Bilateral salpingo-oophorectomy
CCK: Cholecystokinin
CYP7A1: Cholesterol 7α-hydroxylase
DHT: Dihydrotestosterone
ERα: Estrogen receptor alpha
ERβ: Estrogen receptor beta
FGF15: Fibroblast growth factor
FXR: Farnesoid X receptor
GF: Germ-free
GH: Growth hormone
GLP-1: Glucagon-like peptide-1
HDL: High-density lipoproteins
HPA: Hypothalamic–pituitary–adrenal
IFG: Impaired fasting glucose tolerance
IGT: Impaired glucose tolerance
LDL: Low-density lipoproteins
LPL: Lipoprotein lipase
LPS: Lipopolysaccharide
MetS: Metabolic syndrome
miRNA: MicroRNA
NF-κB: Nuclear factor kappa B
NpY: Neuropeptide Y
ODMA: *O*-Desmethylangolensin
PCOS: Polycystic ovary syndrome
POMC: Proopiomelanocortin
PYY: Peptide tyrosine tyrosine
SHBG: Sex hormone binding globulin
SCFAs: Short-chain fatty acids
TNF-α: Tumor necrosis factor alpha
TLR: Toll-like receptor
Treg cells: Regulatory T cells
T2D: Type 2 diabetes
